# Gender Differences in Factors Affecting Life Satisfaction of the Elderly with Multimorbidity in Korea

**DOI:** 10.3390/nursrep11010006

**Published:** 2021-02-01

**Authors:** Jeonghyun Kim, Minkyung Lee, Hyunju Dan

**Affiliations:** 1 College of Nursing, Ewha Womans University, Seoul 03760, Korea; jeonghyunkim@ewha.ac.kr; 2 Infectious Disease Department, Weill Cornell Medicine, New York, NY 10065, USA; mkmk8888@naver.com

**Keywords:** multimorbidity, life satisfaction, gender, elderly, Korea

## Abstract

To enhance the life satisfaction of the elderly with multimorbidity, it is necessary to investigate the relevant factors and to examine the differences in factors according to gender. The aim of this study was to identify factors affecting life satisfaction of the elderly with multimorbidity in the community by gender in Korea. We analyzed data from 2140 participants with multimorbidity who were aged 65 or older and participated in the Korean Longitudinal Study of Ageing (KLoSA) in 2016. A multivariate linear regression analysis was conducted to examine the factors affecting life satisfaction among male and female older participants with multimorbidity. The most common pattern of multimorbidity of women was hypertension and arthritis/rheumatism, while that of men was hypertension and diabetes mellitus. Some factors, such as depression, exercise, and number of chronic diseases, affected both male and female participants, but others related to life satisfaction varied by gender. Therefore, it is necessary to consider the characteristics of multiple chronic diseases, and policy support should be provided in consideration of gender differences to improve the life satisfaction of the elderly.

## 1. Introduction

Multimorbidity, in which a person has two or more chronic diseases simultaneously, has been a concern in the healthcare community, as the elderly population has increased. Multimorbidity prevalence in those aged 65 or older has been reported from 67.5% to 87% worldwide [1,2,3], and multimorbidity tends to increase with age [4]. In Korea, which entered the aging society in 2017, the population aged 65 or older increased to 14.9% of the total population in 2019 [5,6]. Along with the increase in the elderly population, the multimorbidity prevalence was found to be 37.9%–73.0%, which is higher than the prevalence of single chronic diseases of 16.5%–29.2% [2,7], thus management of multimorbidity is necessary.

The patterns of multimorbidity vary depending on research participants and design [7,8], and it has been reported that women are more susceptible to multimorbidity [9,10]. A systematic review study identified that the most common multimorbidity pattern was osteoarthritis with cardiovascular and/or metabolic diseases, but there were differences in patterns according to gender [11].

Compared to people with one chronic disease, the elderly with multimorbidity have increased risk of polypharmacy use [12], functional decline [13], greater health service use and health cost, poor quality of life [3], and increased mortality [14]. Chronic diseases affect one’s attitude toward life, so Pan et al. [15] reported that the number of chronic diseases in the elderly aged 60 or older had a negative effect on life satisfaction. However, it is not well known how elderly people with multimorbidity subjectively evaluate their lives.

Life satisfaction is defined as the overall subjective evaluation of one’s own life [16]. It is important to support the elderly to maintain a high level of life satisfaction, as the elderly with high life satisfaction tend to be more emotionally positive, maintain good health, and have a lower risk of mortality [17,18]. There are various factors that affect life satisfaction in the elderly. Sociodemographic factors include education, marital status, income, employment, health insurance, and participation in religious activities [19,20,21]. Health-related factors such as physical activity, cognition, and depression are also important factors [22,23,24].

In particular, even in people with the same disease, subjective evaluation of one’s health or satisfaction with one’s health can vary, and there might be different health outcomes. A qualitative study that explored the elderly’s perception of their own health found that subjective components involving psychological and social factors had a greater effect on the health evaluation of the participants than did merely their objective health status [25]. Therefore, it is necessary to identify and manage factors affecting the satisfaction with life and health of the elderly who have multiple chronic diseases, but relevant studies are limited.

Because of differences in social norms as well as biological characteristics, different health outcomes may occur depending on gender [26], and life satisfaction may vary. The results of previous studies related to life satisfaction of the elderly have been inconsistent depending on gender. Some studies showed that the level of life satisfaction of older women was higher than that of older men [27,28], while others reported no gender difference in life satisfaction [29].

The results of studies on factors influencing life satisfaction according to gender have not been consistent. Choi and Kim [27] found that monthly income affected life satisfaction of elderly women only, while other researchers [30] reported that income affected the life satisfaction of both genders. A large-scale, multi-country study of people aged 15–99 showed that unemployment and education level affected life satisfaction of both men and women, but affected men more than women [31]. In addition, religion and marital status also affected the life satisfaction of men and women differently [30,31]. Therefore, life satisfaction and affecting factors of the elderly with multimorbidity also are expected to vary by gender. The purpose of this study was to identify the multimorbidity patterns and factors affecting life satisfaction of the elderly with multimorbidity in the community by gender in Korea.

## 2. Methods

### 2.1. Aim and Design

This is a secondary analysis study aimed to identify the determinants of multimorbid older adult life satisfaction by gender using Korean Longitudinal Study of Ageing (KLoSA) data from 2016.

### 2.2. Description of the Data Source, Procedure for Data Collection, and Sampling

The KLoSA is a data-based panel that creates and implements effective social and economic policies to address the emerging trends as the population of Korea ages. This nationally representative survey has been conducted biennially with 10,254 Koreans aged 45 or older, excluding people dwelling in facilities and residents of Jeju Island, from 2006 to 2016 [32]. The survey contains seven domains (population, family, health, employment, income, assets, subjective expectancy, life satisfaction) and addresses the physical and psycho-social factors affecting older peoples’ health status.

The KLoSA data were downloaded from the website of the Korean Employment Survey (https://survey.keis.or.kr). The sixth phase of the KLoSA, which is the most recent data set available for public use, was used to assess the most current trends. Among the 10,254 participants, there were 6618 respondents in the sixth phase, of whom 4522 were older than 65 years. Among the latter, 2140 respondents who had more than two chronic diseases (hypertension, diabetic mellitus, malignant tumor, chronic lung disease, hepatic disease, heart disease, cerebrovascular disease, psychological disease, arthritis, rheumatism) were included in the final analysis. This study was approved by the institutional review board of the researchers’ university (No. 162-13). This is a secondary analysis study aimed to identify the determinants of multimorbid older adult life satisfaction by gender using Korean Longitudinal Study of Ageing (KLoSA) data from 2016.

### 2.3. Study Variables and Measurements

The dependent variable in this study was life satisfaction, and the independent variables were categorized by sociodemographic, health behavioral, and health status factors.

#### 2.3.1. Sociodemographic Factors

Sociodemographic factors included sex (male vs. female); age (years); education level (less than middle school vs. middle school or higher); marital status (married vs. not married; widowed and others); religion (yes vs. no); current employment (yes vs. no); interactions with close friends (more than once a week vs. less than once a week); and private health insurance (yes vs. no).

#### 2.3.2. Health Behavioral Factors

Health behavioral factors were cigarette smoking (never smoked vs. smoker) and alcohol drinking (never drunk vs. drinker); regular exercise was assessed based on yes or no to the question, “Do you exercise more than once a week regularly?”

#### 2.3.3. Health Status Factors

Health status factors comprised number of chronic diseases, cognition, depression, and grip strength. Self-reported diagnoses (hypertension, diabetic mellitus, malignant tumor, chronic lung disease, hepatic disease, heart disease, cerebrovascular disease, psychological disease, and arthritis/rheumatism) were counted as chronic diseases. Cognition was measured using the Mini-Mental State Examination (MMSE) in which the total score is 30 points, with higher scores indicating higher cognition (Korea Employment Information Service, 2018). Depression was measured using the Center for Epidemiological Studies-Depression Scale short-form 10 item (CES-D10). Each item was assessed as 0 = no or 1 = yes, and the score was the sum of the 10 items; a higher score indicated a higher level of depressive symptoms. Grip strength was the mean of two values in kilograms measured for both hands using a grip strength dynamometer. In the previous study, reporting age- and gender- specific distributions of grip strength using data from the Korea National Health and Nutrition Examination Survey, the cut-off values for grip strength in healthy male and female older adults were 28.6 and 16.4 kg, respectively [33].

#### 2.3.4. Life Satisfaction

Life satisfaction was assessed using the question “How satisfied are you with your life compared to that of your peers in the same age group?” in terms of health and overall quality of life (QOL). Participants answered on a scale from 0 to 100 points at intervals of 10 points, with a higher score indicating greater satisfaction. Among the factors of life satisfaction, health and overall QOL were used as dependent variables in this study.

### 2.4. Statistical Analyses

IBM SPSS Statistics 25.0 (IBM, Inc., Armonk, NY, USA) was used for data analysis. Descriptive statistics of frequency, percentage, mean, and standard deviation were used to describe the variables. Differences between older males and females with multimorbidity were analyzed using Chi-square (χ2) and independent t-tests. A multivariate linear regression analysis was conducted to understand the predictors of life satisfaction among older males and females with multimorbidity. Forward selection was used to determine the significant predictors that would be included in the multivariate model. All analysis was conducted by applying population weights calculated to reflect changes in the general distribution of the total population [34].

## 3. Results

### 3.1. Differences between Older Males and Females with Multimorbidity

Table 1 shows the differences in sociodemographics, health behavior, health status, and life satisfaction between older males and females with multimorbidity. Among the 2140 participants, 756 were male with a mean age of 74.17 ± 6.51; 1384 were female with a mean age of 76.22 ± 7.22. The men’s education level was higher than that the women’s; most of the women had been educated to elementary school or less (75.6%), whereas 34.7% of the men had been educated to elementary school or less and 65.3% graduated middle school or higher. Most of the men were married (86.4%), while 46.7% of the women were married. Half of the female participants (51.0%) declared religious belief, in comparison to 36.4% of the males. The male participants who were currently employed numbered 30.6%, while only 13.0% of the female participants were employed. Two-thirds of the women (67.7%) met with close friends more than once a week, compared with 54.8% of males. More men (19.9%) than women (14.2%) had private health insurance.

In terms of health behavioral factors, most male participants were smokers and drinkers (68.9% and 81.2%, respectively), while most of the female participants had never smoked or drunk alcohol (94.0% and 74.7%, respectively). However, more men (40.6%) exercised regularly than did women (25.9%).

Health status factors were number of chronic diseases, cognition, depression, and grip strength. The overall health status of females was lower than that of males; females had a larger number of chronic diseases (2.68 ± 0.87 and 2.52 ± 0.75, respectively); mean MMSE score was 24.98 ± 5.19 in males and 22.31 ± 6.20 in females; females had more depressive symptoms than males (3.84 ± 2.82 and 3.25 ± 2.93); and the mean grip strength of males was higher than that of females (19.29 ± 5.26 and 28.24 ± 7.55).

Male participants were more satisfied with their health and lives than were female participants, with mean health QOL scores of 51.02 ± 20.17 and 47.99 ± 20.49, respectively, and mean overall QOL of 59.18 ± 17.21 and 56.22 ± 17.33.

### 3.2. Differences in Multimorbidity by Gender

**Figure 1:**
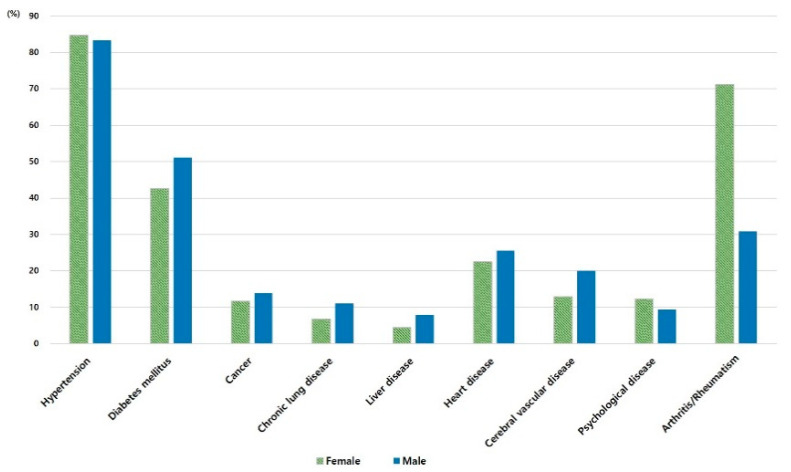
Self-reported diagnosed chronic diseases by gender.

Differences in self-reported diagnosed chronic diseases between older males and females are shown in Figure 1. Hypertension was the most common chronic disease in both females and males (84.7% and 83.3%, respectively). However, arthritis/rheumatism (which was the second-most frequent disease for females) was much more common in females than males (71.2% and 30.8%, respectively). A chronic disease that affected women more than men was psychological disease (12.3% and 9.4%).

Table 2 describes the data of the included 882 females (63.7%) and 438 males (57.9%) to determine the 10 most common combinations of multimorbidity. The most common combination of females was hypertension and arthritis/rheumatism (24.0%), while that of males was hypertension and diabetes mellitus (18.2%). All 10 of the male combinations included hypertension, while six of 10 female combinations involved arthritis/rheumatism.

### 3.3. Predictors of Life Satisfaction in Older Females and Males with Multimorbidity

Table 3 shows the factors influencing satisfaction with health among older people with multimorbidity according to gender. Among female participants, depression, cognition, exercise, number of chronic diseases, current employment, religion, and education were significantly related to satisfaction with health. Among male participants, depression, cognition, exercise, number of chronic diseases, current employment, grip strength, and smoking were significantly associated with satisfaction with health.

The factors influencing satisfaction with QOL among older people with multimorbidity are shown in Table 4. Depression, exercise, number of chronic diseases, religion, cognition, and smoking were the significant factors among females; those of males were depression, exercise, number of chronic diseases, marriage, and private health insurance.

## 4. Discussion

This study analyzed the differences in multimorbidity pattern and life satisfaction according to gender using secondary data of the KLoSA. The combination of hypertension, diabetic mellitus, and arthritis was frequent in both males and females; the most frequent combination of multimorbidity in elderly females was hypertension and arthritis (22.4%), and six of their top 10 combinations included arthritis. In contrast, hypertension and diabetes mellitus was the most frequent combination in elderly males (18.2%); 8.9% of them had hypertension and arthritis, and arthritis was included in two of the top 10 combinations. According to a 2020 report by the World Health Organization, 18.0% of women and 9.6% of men older than 60 years had symptomatic arthritis; 80% of those with arthritis had limitations in movement; and 25% could not perform their major activities of daily living (ADLs) [35].

Arthritis tends to affect elderly females more than it does elderly males, and males performed more regular exercise than did females. Regular exercise is the most significant factor in satisfaction with health and overall QOL. Although pain caused by arthritis is known to interfere with regular exercise [36], proper exercise can relieve pain and improve physical function [37,38]. Therefore, elderly women need to be encouraged to exercise regularly.

In this study, the number of married male elderly was higher than the female elderly. In addition, this study found that elderly men were more satisfied with their health and overall QOL than were women, which is not consistent with the results of several previous studies that elderly women’s life satisfaction level was higher or not significantly different from men’s [27,28]. The average life expectancy of men and women in Korea in 2018 is 79.7 and 85.7 years, respectively, but the life expectancy excluding the disease period is 64.4 years in both males and females, showing that women live longer than men in unhealthy conditions and alone [39]. Therefore, it is necessary to study the change in life satisfaction among the elderly according to age group. In addition, socioeconomic factors such as economic status can affect health status, resulting in gender differences [26]. In a recent study [40], investigating gender differences in QOL among the elderly in low- and middle-income countries, elderly men reported better QOL than women, and income was one of the factors significantly related to QOL. Although South Korea’s annual GDP per capita reached USD$30,000 in 2018, making it a high-income country [41], it has the largest difference in income poverty rates between people aged over 65 (43.8%) and the total population (17.4%) among OECD countries; also, the elderly poverty rate was 49.0% in women and 37.1% in men [42].

Income level was not included in this study, but employment rate and use of private insurance were lower in women than men, indicating worse financial status among women. Since elderly financial status is associated with not only physical and mental health, but also QOL [40,43], gender-specific efforts are required to alleviate poverty in the elderly.

In this study, the factors affecting satisfaction with health and overall QOL in the elderly were different according to gender. Depression, cognition, regular exercise, number of chronic diseases, and employment status were significant factors in satisfaction with health in both elderly women and men, and depression, regular exercise, and number of chronic diseases were significant factors in satisfaction with overall QOL. Multimorbidity most significantly affects satisfaction with health and QOL of both elderly men and women, and these findings were consistent with previous studies [7,44]. However, most healthcare services in Korea are designed to treat single diseases [45]. Therefore, in primary care, management and support for the elderly with multimorbidity need to be provided and improved.

The study analyzed cognition, depression, and grip strength as well as the number of co-morbid diseases as elderly health status factors. These factors influence each other and affect elder life satisfaction. The elderly female participants in this study had more chronic diseases, lower cognition level, and more depression symptoms than the males. These factors significantly affect life satisfaction, which was somewhat consistent with previous studies. Previous studies reported that elderly women’s cognitive function level was lower than that of men’s [46,47] and cognitive capacity was the most influential factor in life satisfaction among both elderly women and men [23,48]. Depression is another important factor that affects elder life satisfaction. Onishi et al. [49] found that depression was a significant determinant of life satisfaction among elderly women, while cognition and comorbidity were not. Similarly, Puvill et al. [50] found that depressive symptoms and loneliness were strongly associated with life satisfaction. Besides, elder grip strength is a major factor that predicts patient nutritional status, physical function, and mortality in previous studies [51] and it is known that grip strength and depression or cognitive capacity have an inverse correlation [52,53]. Therefore, we need to comprehensively approach the physical and psychological factors that influence elder life satisfaction.

In this study, it is difficult to confirm any causal relationships because the cross-sectional design could not confirm the temporal arrangement between variables and all factors that may affect the relationship between variables could not be controlled. The number and type of diseases reported in the study was limited and the severity of multimorbidity was not considered, which may affect life satisfaction in the elderly, so we could not confirm their impacts on life satisfaction. In addition, since this study used secondary data to analyze factors affecting life satisfaction, there were limitations in including the selection of variable measurement tools and other factors that could also affect life satisfaction, such as economic level, family relationship, living arrangement, area of residence, and nutritional status. Therefore, follow-up studies that reflect these factors are suggested. Nevertheless, one of the strengths of this study is that the patterns of multimorbidity and the factors influencing life satisfaction among the elderly are identified by gender using a nationwide sample representing the elderly in Korea.

## 5. Conclusions

This study analyzed the life satisfaction of 2140 elderly men and women with multimorbidity using KLoSa data from 2016. Hypertension and arthritis was the most common combination in women, and hypertension and diabetes mellitus was the most common in men. The factors influencing life satisfaction of both male and female elderly were depression, regular exercise, and number of chronic diseases. Factors affecting the elderly satisfaction with life varied by gender. This study could provide a basis for policy makers to consider gender differences and the characteristics of multimorbidities to improve elder life satisfaction. In addition, since this study focused on the individual factors that affect life satisfaction with health and QOL of the elderly, further research on social factors and systems that affect the health and life satisfaction of the elderly is needed, and should be accessed using the Dahlgren–Whitehead model [54] or Bronfenbrenner’s ecological system [55].

## Figures and Tables

**Table 1:** Differences of variables by gender (*n* = 2140).

Variables		Female (*n* = 1384)	Male (*n* = 756)	*p*
		Weighted %, Mean (*SD*)		
Age (year)		76.22(7.22)	74.17 (6.51)	<0.001
Education	Elementary school or less	75.6	34.7	<0.001
	Middle school or higher	24.4	65.3	
Marital status	Married	46.7	86.4	<0.001
	Not married (widowed, etc.)	53.3	13.6	
Religion	Yes	51.0	36.4	<0.001
	No	49.0	63.6	
Current employment	Yes	13.0	30.6	<0.001
	No	87.0	69.4	
Meeting with close friends	More than once a week	67.7	54.8	<0.001
	Less than once a week	32.3	45.2	
Private health insurance	Yes	14.2	19.9	<0.001
	No	85.8	80.1	
Cigarette smoking	Never smoked	94.0	31.1	<0.001
	smoker	6.0	68.9	
Alcohol drinking	Never consumed	74.7	18.8	<0.001
	Has consumed	25.3	81.2	
Regular exercise	Yes	25.9	40.6	<0.001
	No	74.1	59.4	
Number of chronic diseases		2.68 (0.87)	2.52 (0.75)	<0.001
Cognition		22.31 (6.20)	24.98 (5.19)	<0.001
Depression		3.84 (2.82)	3.25 (2.93)	<0.001
Grip strength		19.29 (5.26)	28.24 (7.55)	<0.001
Satisfaction of life	Health	47.99 (20.49)	51.02 (20.17)	<0.001
	Overall quality of life	56.22 (17.33)	59.18 (17.21)	<0.001

**Table 2:** The most frequent multimorbidity type by gender.

	Female	*n* (%)	Male	*n* (%)
1	HTN, AR	333 (24.0)	HTN, DM	138 (18.2)
2	HTN, DM, AR	168 (12.1)	HTN, AR	68 (8.9)
3	HTN, DM	118 (8.5)	HTN, HD	49 (6.4)
4	HTN, HD, AR	59 (4.2)	HTN, DM, HD	40 (5.2)
5	DM, AR	47 (3.4)	HTN, DM, AR	35 (4.6)
6	HTN, HD	45 (3.2)	HTN, CVD	34 (4.5)
7	HTN, DM, HD, AR	36 (2.6)	HTN, Cancer	26 (3.4)
8	HTN, DM, HD	27 (1.9)	HTN, DM, CVD	19 (2.5)
9	HTN, CVD	25 (1.8)	HTN, CLD	16 (2.1)
10	HTN, CVD, AR	24 (1.7)	HTN, DM, Cancer	13 (1.7)

Note. HTN = hypertension; AR = arthritis/rheumatism; DM = diabetes mellitus; HD = heart disease; CVD = cerebral vascular disease; CLD = chronic lung disease.

**Table 3:** Factors affecting life satisfaction with health by gender (*n* = 2140).

Variables	Female (*n* = 1384)				Male (*n* = 756)			
	B	S.E.	β	*p*	B	S.E.	β	*p*
Depression	−2.286	0.182	−0.316	<0.001	−1.967	0.236	−0.286	<0.001
Cognition	0.487	0.085	0.148	<0.001	0.549	0.137	0.141	<0.001
Exercise (ref. No)	5.790	1.158	0.124	<0.001	5.530	1.325	0.135	<0.001
Number of chronic diseases	−2.868	0.557	−0.122	<0.001	−3.565	0.839	−0.133	<0.001
Current employment (ref. No)	3.859	1.465	0.063	0.009	3.373	1.463	0.077	0.021
Religion (ref. No)	2.526	0.973	0.062	0.010				
Education (ref. Elementary school or less)	2.309	1.155	0.048	0.046				
Grip strength					0.309	0.094	0.116	0.001
Smoking (ref. No)					−2.998	1.346	−0.069	0.026
	Adjusted R^2^ = 0.249, F = 66.650 (*p* < 0.001)				Adjusted R^2^ = 0.290, F = 45.026 (*p* < 0.001)			

**Table 4:** Factors affecting life satisfaction with overall quality of life by gender (*n* = 2140).

Variables	Female (*n* = 1384)				Male (*n* = 756)			
	B	S.E.	β	*p*	B	S.E.	β	*p*
Depression	−2.587	0.151	−0.422	<0.001	−2.349	0.189	−0.400	<0.001
Exercise (ref. No)	4.052	0.960	0.102	<0.001	5.811	1.092	0.166	<0.001
Number of chronic diseases	−1.878	0.468	−0.094	<0.001	−2.253	0.709	−0.099	0.002
Religion (ref. No)	2.108	0.814	0.061	0.010				
Cognition	0.165	0.069	0.059	0.017				
Smoking (ref. No)	−3.888	1.689	−0.053	0.021				
Marriage (ref. No)					6.678	1.563	0.133	<0.001
Private health insurance (ref. No)					4.594	1.333	0.107	0.001
	Adjusted R^2^ = 0.261, F = 82.618 (*p* < 0.001)				Adjusted R^2^ = 0.294, F = 63.784 (*p* < 0.001)			

## Data Availability

Publicly available datasets were analyzed in this study. This data can be found here: http://survey.keis.or.kr/klosa/klosadownload/List.jsp.
